# Recurrent Exercise-Induced Rhabdomyolysis in a Healthy Adolescent Girl

**DOI:** 10.7759/cureus.11462

**Published:** 2020-11-12

**Authors:** Nicolas C Longobardi, Yen Longobardi

**Affiliations:** 1 Medicine, St. George's Hospital Medical School, London, GBR; 2 Pediatrics, Yen Longobardi, M.D, Rhode Island, USA

**Keywords:** cardiomyopathy, creatine kinase, rhabdomyolysis, exertional rhabdomyolysis, duchenne muscular dystrophy (dmd)

## Abstract

Female symptomatic carriers of Duchenne muscular dystrophy (DMD) are uncommon findings. Much of this disease has been studied from a male perspective, but female disease presentation and progression are rarely described. This report describes a female adolescent patient with a rare and unconventional presentation of DMD.

## Introduction

Duchenne muscular dystrophy (DMD) is a genetic disorder that is characterized by progressive muscle weakness and degeneration. It is the most common and severe form of muscular dystrophy in the world, having a prevalence of approximately six per 100,000 individuals in Europe and North America [1]. DMD is caused by pathogenic variants in the DMD gene, which encodes the production of the structural protein dystrophin found in the brain, skeletal muscle, cardiac muscle, and retina. Dystrophin plays an important role in maintaining the integrity of the muscle membrane [2]. As DMD is inherited in an X-linked recessive manner, it usually only affects males. However, in rare instances, females are also affected. Approximately 2.5% to 7.8% of female carriers of DMD manifest symptoms to some extent, ranging from mild muscle weakness to rapidly progressive dystrophy and gait problems [3-4]. Patients with DMD also often have a family history of the disease. 

## Case presentation

A previously healthy 14-year-old female presented to the office with sharp, generalized abdominal pain and dark brown urine since the previous night. Additionally, the patient noticed that her whole body ached after running for two hours that same day. She was afebrile and had a cough and runny nose for the past three days. All of these symptoms were not preceded by trauma or *Streptococcus* infection within the past month. Her last menstrual period was 20 days earlier.

On observation, the patient looked well. All vital signs were normal. Physical examination findings were unremarkable (non-tender abdomen, no edema noted on extremities). Urine sampling demonstrated light yellow clear urine. A urine dipstick was not performed because it was unavailable in the office. Consequently, the patient was advised to call the office or go to the emergency room should she have the reemergence of abdominal pain or brown urine. Shockingly, the office was notified that the patient had been admitted to a hospital with acute renal failure the following day. She was in the hospital for three days before finally being discharged home to be followed up by the renal clinic.

Four months later, the patient presented to the office with diffuse back pain of one day's duration. The pain did not radiate anywhere. She also presented with chest pain that had been going on for five months. The pain occurred mainly in the left lower breast area, and it typically increased with inspiration. It occurred roughly one to three times per week and, along with a cough, was associated with recreational sports. The patient stated that the last time she had any chest pain was the previous week. An electrocardiogram (EKG) taken four months earlier was unremarkable. She denied any palpitations, nausea, vomiting, and any dizziness during exercise or rest. Upon further questioning, it was discovered that her throat had felt ‘tight’ for the past year when running, and she often tried to cough because it hurt to swallow. This did not negatively affect her ability to participate until the end of the run, however. In general, she didn’t experience any fever, changes in weight, muscle weakness, or swelling of extremities. She denied having any episodes of dark brown urine ever since being discharged from the hospital. Consequently, the patient was diagnosed with exercise-induced asthma and back muscle strain. 

At a follow-up visit four days later, the patient felt well, but her creatine phosphokinase (CPK) was 2,439 U/L (reference range: 26.0 - 192.0 U/L). This was observed even eight days after she played kickball for one hour. At that time, she was advised to avoid sports and to do a weekly CPK test for three weeks. Table 1 shows the results.

**Table 1:** Creatine Phosphokinase (CPK) Test Results 03/14/2017 values were recorded when the patient was admitted to the hospital for acute renal failure. Four months prior, the patient was diagnosed with dehydration due to strenuous exercise and lack of fluid intake at a clinic (which was disclosed at the last office follow-up). 08/01/2017 correlates to eight days after one hour of kickball 08/04/2017 - 08/23/2017: The patient stopped sports but occasionally played with younger siblings and jumped on a trampoline for about 15 minutes at a time CK: creatine kinase; CK-MB: creatine kinase-myocardial band; N/A: not available

Component name	08/23/2017	08/14/2017	08/04/2017	08/01/2017	03/14/2017
Total CK (U/L) (normal range: 26.0 - 192.0 U/L)	6,899	8,354	3,049	2,439	> 20,000
CK-MB (ng/mL) (normal range: 0.5 - 3.6 ng/mL)	44.7	33.2	23.7	N/A	N/A
Troponin I (ng/mL) (normal range: 0 - 0.4 ng/mL)	N/A	0.029	N/A	N/A	N/A

On further questioning, the patient disclosed that eight months earlier, she had presented to a clinic with dark brown blood in the urine of three days duration (four days after the completion of her menses). The patient reported starting a strenuous exercise regimen on the day she started seeing the brown urine and had generalized exercise-related soreness. She mentioned having been in school sports (mainly basketball), which she had been doing for three years now. The basketball practice and games lasted up to a maximum of two hours, with the first 15 to 30 minutes generally being running. She did not participate in any weightlifting. On average, she drank two bottles of water and half a bottle of Gatorade a day. Urine dipsticks showed the presence of protein and blood in the urine. She was sent home with a diagnosis of dehydration due to strenuous exercise and not drinking enough fluids.

The patient’s past medical history was unrevealing. Occasionally, she took acetaminophen for pain as needed. The patient had been performing really well in school, honoring in all of her classes. She denied any smoking, alcohol consumption, or recreational drug usage. Her mother has attention deficit hyperactivity disorder (ADHD), hypertension, and arrhythmias. Her maternal grandfather suffered from a heart attack, and her maternal grandmother had melanoma three times. One of her second cousins suffered a cardiac arrest and was found to have a prolonged QTc (it was unclear whether this developed before or after the cardiac arrest). Her half-brother has an autism spectrum disorder and ADHD. Her full biological brother is very healthy.

At the time of the follow-up visit in the office, the patient weighed 132 lbs (59.9 kg, 74th percentile) and was 67 inches tall (1.7 meters, 92nd percentile). Her percentiles for weight and height have been the same since the age of three. Her body mass index (BMI) was 20.7 (62nd percentile). Muscle tone, strength, and mass were normal. There were no signs of calf muscle hypertrophy. Gower’s sign was negative (which was performed because the CPK levels were greater than 100 times the upper normal limit when she was previously hospitalized). In the hospital, the following were also unremarkable: CT scan, antineutrophil cytoplasmic antibodies (ANCA), C3/C4 complement, antistreptolysin O titers, *Streptococcus A* direct probe, and a pregnancy test. Erythrocyte sedimentation rate and liver function tests were also normal. Table 2 compares various measurements taken at the time of hospitalization to the time of follow-up.

**Table 2:** Serum Laboratory Values * indicates a high value Urine dipstick on 03/14/17 demonstrated straw-colored urine with 2+ blood and 30 mg/dL of protein. Microscopic urinalysis: RBCs < 1/HPF, WBC 2/HPF, and no casts reported. BUN: blood urea nitrogen; CO_2_: carbon dioxide; HPF: high power field; N/A: not available; RBCs: red blood cells; WBC: white blood cells

Serum Labs	03/14/17 (hospitalized)	08/04/2017 (follow-up in office)
Glucose (mg/dL)	88	88
BUN (mg/dL)	33*	11.0
Creatinine (mg/dL)	4.99*	0.61
Sodium (mEq/L)	134	139
Potassium (mEq/L)	4.9	4.3
Chloride (mEq/L)	104	103
CO_2_ (mEq/L)	23	30.0
Calcium (mg/dL)	9.3	9.2
Magnesium level (mEq/L)	2.1*	N/A
Phosphorus level (mg/dL)	4.2	N/A

The patient’s history of recurrent rhabdomyolysis related to sports, acute renal failure, absence of weakness, and persistently elevated CPK levels with low exercise threshold raised suspicion for metabolic myopathy. She was consequently referred to a geneticist for evaluation. The geneticists performed lactic acid and acylcarnitine panels, both of which were normal. Whole exome sequencing (WES) revealed a pathogenic partial gene deletion in the DMD gene. Pathogenic variants in this gene are associated with Duchenne/Becker muscular dystrophy (DMD/BMD). This was surprising because DMD is X-linked. It was traditionally thought that only males developed this condition.

On recent subsequent evaluations, she tended to present with calf muscle pain and cramping during exercise, muscle twitching all over (although mostly on extremities), and weakness with climbing and trying to get up from a chair. She was still able to participate in sports after school with frequent drinking and resting as needed. She was advised to drink enough fluids and monitor her urine color so that it remained clear.

## Discussion

This patient is considered to be a manifesting carrier of DMD with recurrent exertional rhabdomyolysis. Due to the fact that recurrent rhabdomyolysis has many different life-threatening causes, WES was indicated in order to analyze the most related genes in the genome. This has been identified as an efficient way to diagnose and manage DMD patients [5]. WES of the patient described in this report revealed that she was heterozygous for a de novo partial gene deletion in the DMD gene (mother and father did not have this). There are two possible explanations for this finding: either the genetic mutation has existed for generations without anyone knowing, or the mutation arose in the mother’s egg cell [6]. The latter would explain why the standard testing would not be able to pick this up - the mutation would not present in the mother’s blood. Deletions in one or more exons is the most common type of mutation; it is found in approximately 70% of cases of Duchenne [7].

Although there is no precise definition, exertional rhabdomyolysis (ER) is often described as a syndrome characterized by muscle injury associated with strenuous exercise [8]. Athletes are commonly affected, and most cases resolve spontaneously without any symptoms or complications. Although relatively uncommon and seemingly benign, it is important to recognize and appropriately manage ER since it can potentially result in a fatal outcome; it represents the most common cause of acute kidney injury and acute renal failure in athletes [9-10]. Similar to this patient, patients with ER typically present with muscle pain, elevated levels of serum creatine kinase, and ‘cola-colored’ urine [10].

When a muscle is damaged, it releases potentially toxic intracellular contents into the plasma, such as potassium, phosphate, myoglobin, and creatine kinase. Myoglobin appears in the urine when the plasma concentration of myoglobin exceeds 1.5 mg/dL, but urine dipstick may be negative in up to half of patients with rhabdomyolysis [11-12]. Urine color does not visibly change until the myoglobin urine concentration exceeds about 100 mg/dL to 300 mg/dL [13]. Myoglobin is rapidly cleared from the serum into the urine within 24 hours of symptom onset, which is the reason why the patient’s urine color was light yellow in the clinic and in the office [8]. The patient’s diagnosis of acute kidney injury (AKI) can be explained by the fact that myoglobin is nephrotoxic: it produces renal vasoconstriction, is directly cytotoxic (due to its heme protein), and large amounts obstruct renal tubules [14]. Interestingly, another study also showed that the receptors involved in the renal reuptake of myoglobin are the same receptors involved in nephrotoxicity [15].

Serum creatine kinase (CK) is the most reliable diagnostic marker of rhabdomyolysis. Patients with ER often present with drastically elevated serum CK levels, sometimes as high as 50 times the upper limit of normal [8]. In patients with rhabdomyolysis, serum CK levels typically rise within 12 hours after muscle injury, peak within one to three days, and decline three to five days after [11]. Its half-life is approximately 36 hours, and the body clears roughly 40% - 50% of the previous day’s levels. Peak CK levels are one of the many determinants of renal failure. The creatine kinase-muscle (CK-MM) isoform generally constitutes all, if not the majority, of the CK released as a result of rhabdomyolysis. The creatine kinase-myocardial band (CK-MB) isoform may constitute a small portion of the released CK. Interestingly, a study showed that serum myocardial band (MB) levels in DMD levels reflected a skeletal muscle origin rather than cardiac muscle [16].

The rarity surrounding this case - a female manifesting carrier of DMD - renders it easy to overlook. Many physicians would not expect females to have DMD, much less recognize the potential presentations. One study reported two female carriers with dilated cardiomyopathy, elevated serum CK levels, and absence of muscle weakness. Thus, it was suggested that dilated cardiomyopathy may be the only manifestation of dystrophin mutation in carriers [17]. On the contrary, another more recent study, which included four manifesting female carriers, reported all of them having muscle weakness and elevated CK levels [18]. Another study suggested that female carriers of DMD develop muscle weakness about 17% of the time and approximately 33% present with cardiac or cognitive abnormalities. Its investigation of female DMD carriers revealed that scoliosis, calf hypertrophy, and myopathic patterns at electromyography may help recognize pediatric DMD carriers [19]. It has often been observed that swallowing difficulties can pose a challenge to patients with DMD [20]. The patient experienced such difficulties occasionally and only during exercise.

## Conclusions

In conclusion, our patient represents a unique case of a symptomatic female DMD carrier presenting with recurrent exertional rhabdomyolysis. This case highlights the importance of recognizing that pathology may present with wide variability. Assumptions based on typical clinical criteria (e.g., male gender in DMD) can lead to misdiagnosis. This patient has been followed by cardiology and will be followed by a neuromuscular clinic for further monitoring of her disease. 
